# EEGNET: An Open Source Tool for Analyzing and Visualizing M/EEG Connectome

**DOI:** 10.1371/journal.pone.0138297

**Published:** 2015-09-17

**Authors:** Mahmoud Hassan, Mohamad Shamas, Mohamad Khalil, Wassim El Falou, Fabrice Wendling

**Affiliations:** 1 INSERM, U1099, Rennes, France; 2 Université de Rennes 1, LTSI, France; 3 Lebanese University, AZM Center for Biotechnology Research and Its Applications, Tripoli, Lebanon; University of Minnesota, UNITED STATES

## Abstract

The brain is a large-scale complex network often referred to as the “connectome”. Exploring the dynamic behavior of the connectome is a challenging issue as both excellent time and space resolution is required. In this context Magneto/Electroencephalography (M/EEG) are effective neuroimaging techniques allowing for analysis of the dynamics of functional brain networks at scalp level and/or at reconstructed sources. However, a tool that can cover all the processing steps of identifying brain networks from M/EEG data is still missing. In this paper, we report a novel software package, called EEGNET, running under MATLAB (Math works, inc), and allowing for analysis and visualization of functional brain networks from M/EEG recordings. EEGNET is developed to analyze networks either at the level of scalp electrodes or at the level of reconstructed cortical sources. It includes i) Basic steps in preprocessing M/EEG signals, ii) the solution of the inverse problem to localize / reconstruct the cortical sources, iii) the computation of functional connectivity among signals collected at surface electrodes or/and time courses of reconstructed sources and iv) the computation of the network measures based on graph theory analysis. EEGNET is the unique tool that combines the M/EEG functional connectivity analysis and the computation of network measures derived from the graph theory. The first version of EEGNET is easy to use, flexible and user friendly. EEGNET is an open source tool and can be freely downloaded from this webpage: https://sites.google.com/site/eegnetworks/.

## Introduction

Magneto/Electroencephalography (M/EEG) are key techniques to analyze functional connectivity from surface signals [1, 2] or/and from reconstructed brain sources [3, 4]. The main advantage of M/EEG is the excellent temporal resolution (sub-second) that offers the unique opportunity i) to track brain networks over very short duration which is the case in many cognitive tasks and ii) to analyze fast dynamical changes that can occur in brain disorders (like epileptic seizures for instance).

So far, approaches based on graph theory have represented brain networks as sets of nodes interconnected by edges [5]. Once the nodes and edges are defined from the neuroimaging data, algorithms based on graph theory can be applied to measure the topological properties of considered networks. The application of these algorithms on functional, as well as on structural connectivity matrices, have revealed many properties of brain networks, such as small-worldness [6, 7], modularity [8, 9], hubs [10] and rich-club configurations [11].

The graph theory based analysis has been widely used to characterize normal [12] and pathological [13] brain activities from several modalities. It has been used in many applications such as aging [14–16], Alzheimer’s disease [17–20], epilepsy [21–23], schizophrenia [24, 25] and autism [26].

In the M/EEG context, nodes represent either the electrodes or the dipole sources depending on whether the connectivity is analyzed at scalp or at reconstructed source level, respectively. The edges are defined by the values of the statistical dependencies among M/EEG signals or among reconstructed time courses of cortical sources.

On the one hand, several tools were developed to process M/EEG signals such as EEGLAB [27], CARTOOL [28], Fieldtrip [29] and Brainstorm [30]. On the other hand, many other tools have been proposed to analyze and visualize complex networks such as Brain Connectivity Toolbox (BCT) [31], BrainNet Viewer [32], the GCCA toolbox [33], the connectome mapper [34], Gephi [35], the connectome Viewer [36], the eConnectome [37], the Connectome Visualization Utility (CVU) [38] and GraphVar [39].

All these packages are typically specialized for processing a particular step in the whole pipeline aimed to identifying and characterizing brain networks. However, a tool that comprises the complete pipeline from M/EEG processing to analysis/visualization of brain networks is still missing. This consideration led us to develop and present EEGNET, MATLAB-based software with Graphical User Interface (GUI). Our main objective was to develop a complete framework that can cover most of the processing from EEG recordings to graph analysis and visualization. This pipeline includes: 1) loading and filtering the M/EEG signals, 2) the solution to the inverse problem and the reconstruction of the cortical sources, 3) the computation of the functional connectivity, 4) the calculation of the network measures and 5) the visualization of 2D (scalp level) and 3D (cortex level) brain networks and associated measures.

## Methods and Results

**Fig 1:**
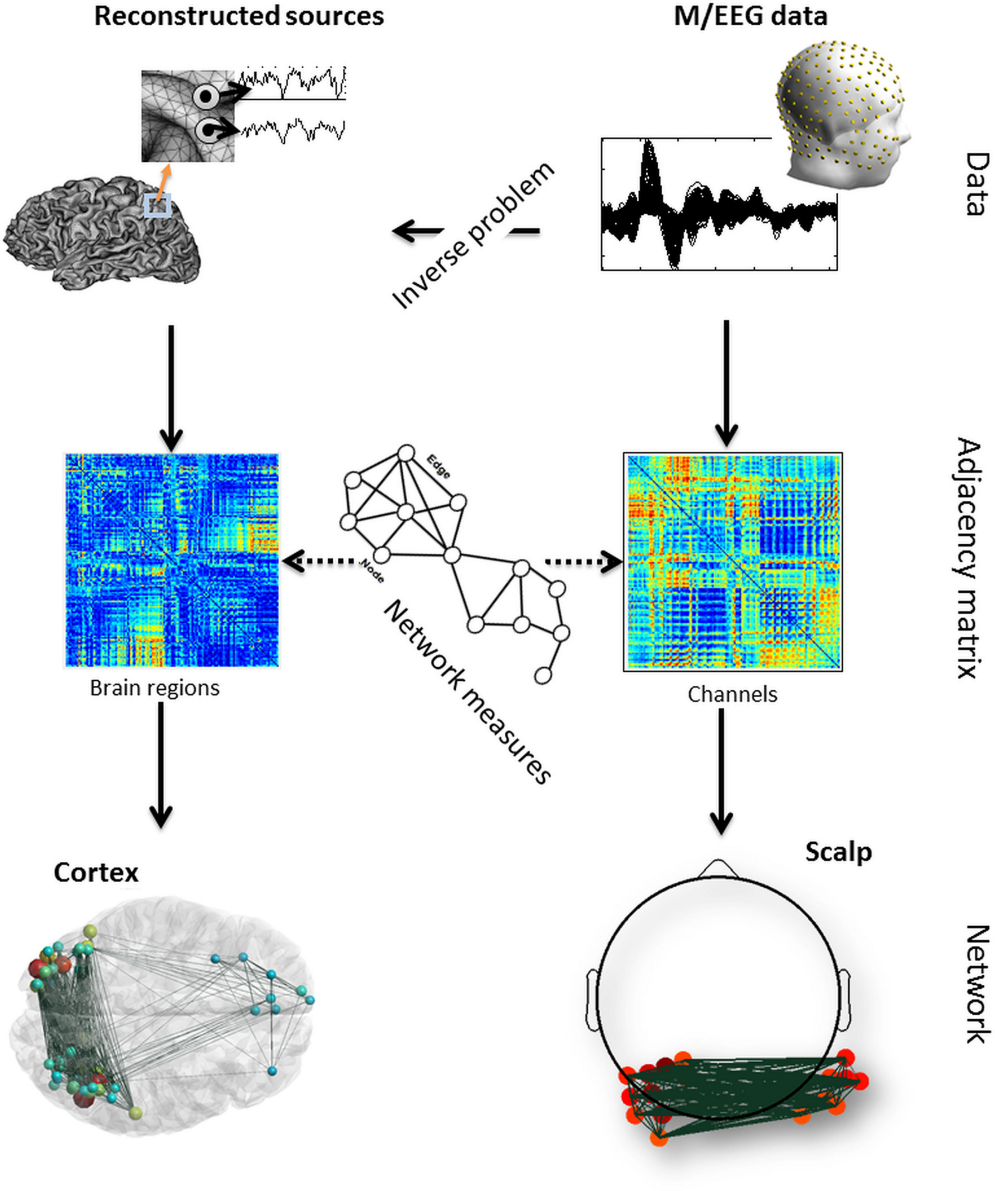
Basic workflow of EEGNET. The M/EEG data are imported (256 dense EEG signals is this example). The functional connectivity is then computed directly between the scalp signals. Network measures can be extracted from the adjacency matrix and the scalp network can be visualized in an interactive way. On the other hand, the original M/EEG data can be used to estimate the brain sources by solving the inverse problem. Functional connectivity measures can be applied on the reconstructed sources. Graph measures can also be computed and the correspondent cortex network can be visualized. Node’s size and color can be used to encode any chosen network measures (their strength for instance) as well as the edges (their weight for instance).

EEGNET is a useful processing pipeline to identify, visualize and characterize brain networks from M/EEG recordings. It can perform all steps including the estimation of brain sources, the computation of the functional connectivity and the mapping of brain networks at scalp level and/or at source level. The basic workflow is shown in Fig 1.

### Overview

The main elements of EEGNET are:

#### The data

This file represents either the scalp EEG data or the reconstructed sources. The default file format is the ‘.mat’. It should be a 3 dimensional matrix (*Nc* x *Ns* x *Nt*) where *N*_*c*_, *N*_*s*_ and *N*_*t*_ are the channels (brain regions in the case of sources file) number, the sample size and the number of trials (*N*_*t*_ is considered 1 for data averaged over trials), respectively. When solving the inverse problem and for visualizing the network at scalp level, the electrode location file is required. In the current EEGNET version, both the.*xyz* and.*mat* formats are supported.

Some basic preprocessing features are available in the current version of EEGNET. The imported data can be firstly visualized. These signals can be then filtered using a Finite Impulse Response (FIR) linear filter before computing the function connectivity (FC) matrices. This feature allows users to choose the frequency band where to compute the FC. The data can also resampled by changing the sampling frequency. This feature can be essential in some cases where users attempt to reduce the size of the data. The preprocessing tool allows user also to specify the baseline from the visualized data. This baseline is essential for computing/normalizing the post vs. pre stimulus connectivity for instance or to compute the noise covariance matrix when solving the inverse problem.

EEGNET provides also the possibility of computing the time-frequency representation of the data. In the current version, the complex Morlet wavelet is used as it was shown to provide a good compromise between time and frequency resolution [40–42]. This time frequency maps can be shown trial by trial in the case of multi-trial data.

#### The adjacency matrix

This file is an *Nc* x *Nc* dimension. It contains the values of the functional connections between all the channels (or brain regions). This file can be also in *Ns* x *Nc* x *Nc* in the case where it is the dynamics of functional networks that is being analyzed. To compute the functional connectivity (FC) matrices, four methods are available: the cross-correlation, the mean phase coherence (MPC), the mutual information (MI) and the Phase Locking Value (PLV), see [43] for review. After choosing the desired method, the connectivity values can be computed over scalp signals (generating 2D networks) or over the time series associated with the reconstructed sources (generating 3D networks at cortex level). In the typical example presented in this paper, the Phase Locking Value (PLV) was computed between scalp electrodes as well as between sources. The PLV is a part of the method from PS family. It was initially proposed by Lachaux et al. [44] and its main advantage is the possibility of computing FC matrix at each instant as the method look at the inter-trial information [2]. To assess the significance of the obtained connections, surrogates data analysis can be used and a level of significance can be set which allow users to keep only the statistically significant connections (see [2] for details about this approach).

To ensure the significance of the obtained FC matrices, we integrated a statistical test based on the surrogate data analysis. Briefly, we use multivariate Fourier transform surrogates generated from the original EEG data. Such surrogates correspond to realizations of linear stationary process with conserved auto-and cross-correlation characteristics. The null hypothesis is tested by comparing the original connectivity value (C_org_) and those obtained using the surrogate data (C_surr_) using a statistical test. The “Rank test” is used to reject or accept the null hypothesis. Basically, [C_org_; C_surr_] is sorted in increasing order and the rank index for C_org_ is returned. With a number of surrogates (n_surr_ = 100 for example), if this rank is > 95 and < 5 (significance level at 95%), this means that it lies in the tail of the distribution, and that the null hypothesis can be rejected (two-tailed test) with a significance of p = 2*(1/ (n_surr_+1)) = 0.019. The output of this analysis is the matrix containing only the significant connections.

#### The networks

When realizing M/EEG source connectivity, the cortical surface file and its corresponding scout file are required. The surface file contains the cortical mesh and the scout file contains the labels of the brain regions in case of using specific atlas. The cortical parcellation provides the ROIs that are used as network nodes in EEGNET. The surface files store geometric information about the morphology of the cortex. It can be created using the open source imaging analysis tool FreeSurfer http://surfer.nmr.mgh.harvard.edu/. The surface file can be also checked using brainstorm http://neuroimage.usc.edu/brainstorm/. The identification of the ROIs in the matrices is determined by a scout file which is a.mat file (can be generated also using Brainstorm). This file contains the labels of all the ROIs based on the already used atlas for segmentation such as Desikan [45] and Destrieux [46].

To characterize the obtained networks, graph theory based analysis was widely used and proved its high performance and usefulness [31]. A graph is a simple model of a system that are based on a set of nodes (electrodes or brain regions in our case) and the edges between them (functional connectivity values). Using EEGNET, several graph metrics can be computed and can be divided into three categories:

A. Global features

- *Density*: the density of a graph is the fraction of present edges to all possible connections. If the density of a graph is 1 then it is a complete graph (every vertex is connected to every other vertex)
- The *characteristic path length*: is the average shortest path lengths in the network
- The *global efficiency* is the average inverse shortest path length in the network
- *Radius*: The radius of a graph is the minimum graph eccentricity of any graph vertex in a graph. A disconnected graph therefore has infinite radius. Eccentricity is the maximum graph distance between a vertex v and any other vertex u of the graph.
- *Diameter*: graph's diameter is the largest number of vertices which must be traversed in order to travel from one vertex to another.

B. Node parameters

- *Degree*: Node degree is the number of links connected to the node. In case of directed graph, the indegree is the number of inward links and the outdegree is the number of outward links, and the total degree is the sum of both indegree and outdegree.
- *Clustering Coefficient*: The clustering coefficient is the fraction of triangles around a node i.e. the fraction of node’s neighbors that are neighbors of each other.
- *K-Coreness Coefficient*: The k-core is the largest subgraph comprising nodes of degree at least k. The coreness of a node is k if the node belongs to the k-core but not to the (k+1)-core.
- *Node Betweenness*: Node betweenness centrality is the fraction of all shortest paths in the network that contain a given node. Nodes with high values of betweenness centrality participate in a large number of shortest paths.
- *Participation Coefficient*: Compares the number of links (degree) of node i to nodes in all clusters with its number of links within its own cluster. The participation coefficient plays an important role in classifying the nodes of a graph as connector hubs and provincial hubs.

C. Edge parameters

- *Edge Betweenness*: Edge betweenness centrality is the fraction of all shortest paths in the network that contain a given edge. Edges with high values of betweenness centrality participate in a large number of shortest paths.
- *Shortcut Edges*: Shortcuts are central edges which significantly reduce the characteristic path length in the network.
- *Edges Neighborhood Overlap*: is the number of nodes that are neighbors of the nodes of that edge.

See [31] for details and equations of the mentioned and other graph metrics.

### Visualization

#### Scalp level

**Fig 2:**
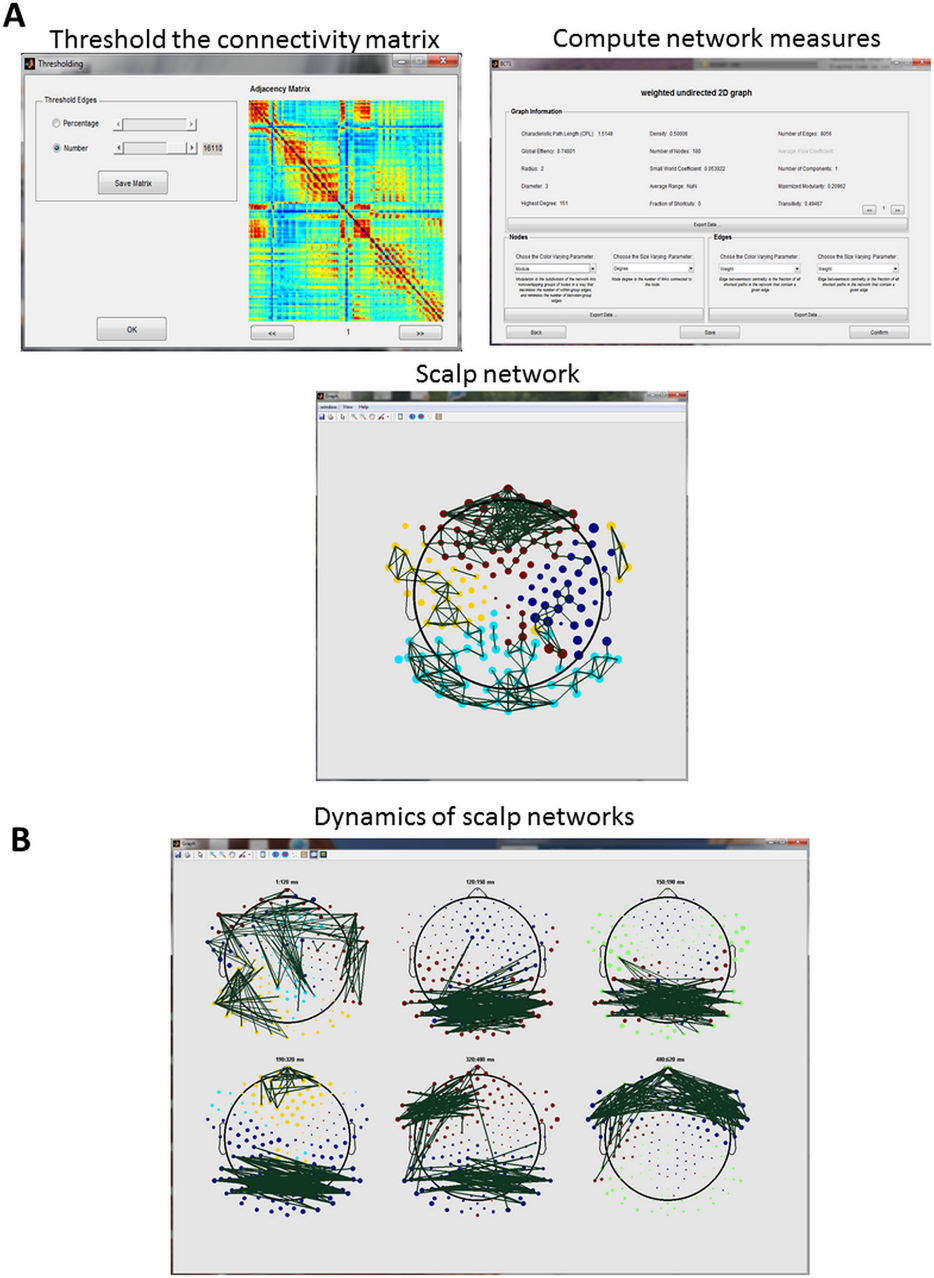
Scalp networks. A) The different steps performed to obtain a ‘static’ scalp network, B) typical example of the dynamics of scalp networks obtained during a picture naming task (see [47] for about the data). The node color represents the modules and the size represents the strength values.

When the user is only interested in scalp networks, the EEG data should be firstly loaded. The functional connectivity is then computed among scalp signals, according to a pairwise procedure. For visualizing the network and computing the network measures, the channel file is required. Users can also directly import their connectivity matrices (computed elsewhere) to compute the network measures and visualizing the network. The channels position is a file with four columns, the first for the node number or label, the next three for *x*, *y* and *z* positions (an example of channel location file is contained within in the *Examples* folder included in the downloaded EEGNET). This part supports the static and dynamic option. The static option requires a 2D matrix *(Nc* x *Nc)* while the dynamic behavior option requires 3D matrices *(Ns* x *Nc* x *Nc)*. Typical examples of the static and dynamic scalp networks are presented in Fig 2A and 2B respectively.

Fig 2A shows the main steps after loading the connectivity matrix and its thresholding process, computing the network measures and visualizing the scalp network. The nodes size and color are used to encode any of the chosen features such as the modules for the color and the strength for the size as presented in the Fig 2A. Fig 2B shows the ability of EEGNET to display the dynamics of the functional networks at different time windows. The data used in this example are from the same cognitive task (picture naming) as the one used in [47] and available on the EEGNET webpage. It shows the tracking of functional scalp networks from the presentation of a visual stimulus to the naming process.

#### Cortex level

To compute the brain networks at source level from M/EEG data, the inverse problem must be solved. It consists of reconstructing the brain sources from the scalp M/EEG. When the M/EEG signals are checked and approved for further analysis, the time series of the reconstructed sources can be estimated. After loading the coordinates of the electrodes as well as a brain surface mesh, the lead field matrix can be computed using different tools such as ‘OpenMEEG’ [48]. The time courses of the sources are then estimated by solving the inverse problem. Several algorithms for solving the inverse problem can be used (see [49] for review). In the example showed here, the weighted Minimum Norm Estimate (wMNE) was used [50].

**Fig 3:**
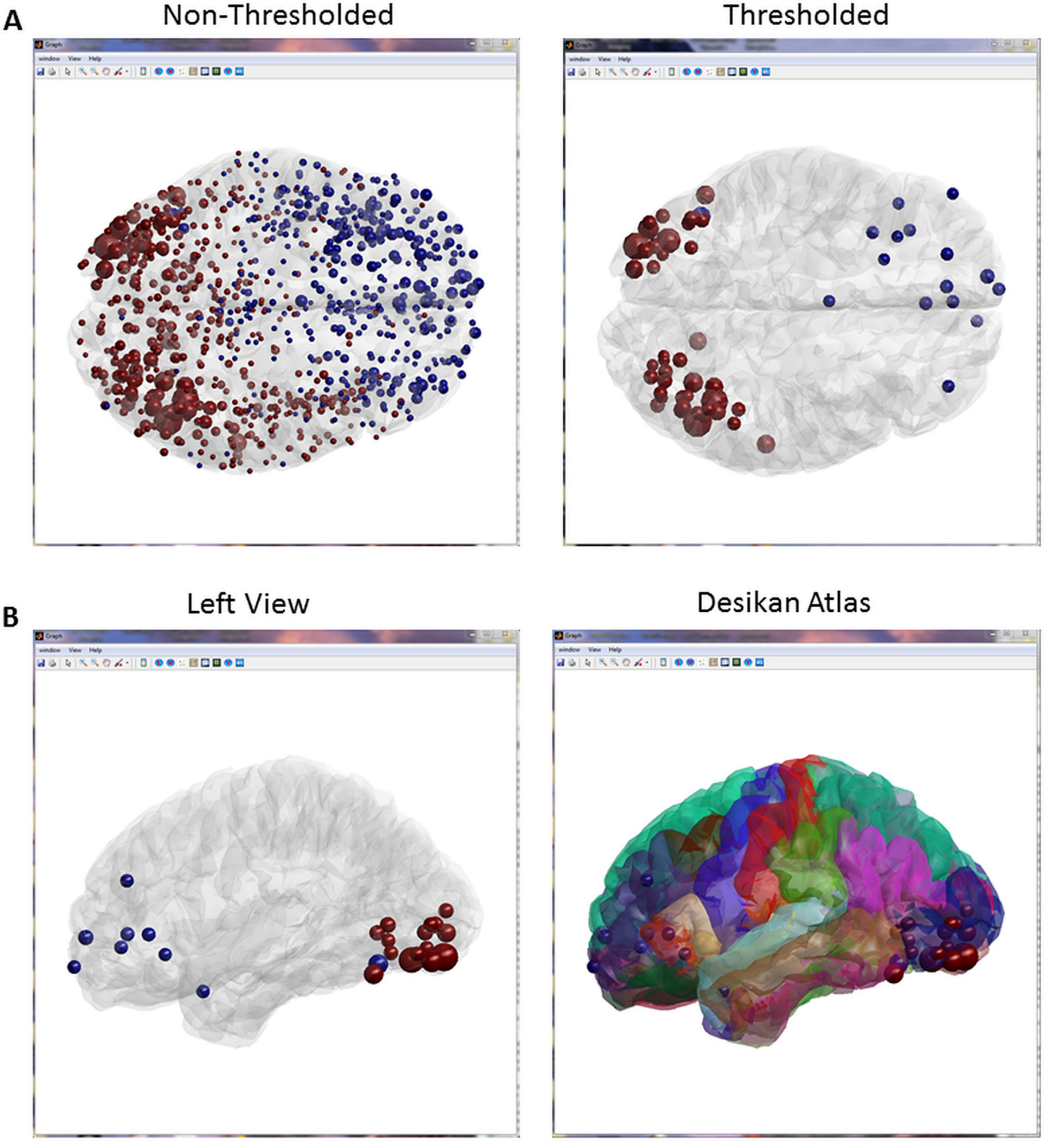
Example of the nodes visualization control. A. All nodes are showed with and without thresholding (about 1000 ROIs). Node’s color represents the module and nod’s size represents the strength values. B. The left view of the same network with the corresponding Desikan Atlas imported from scout file.

**Fig 4:**
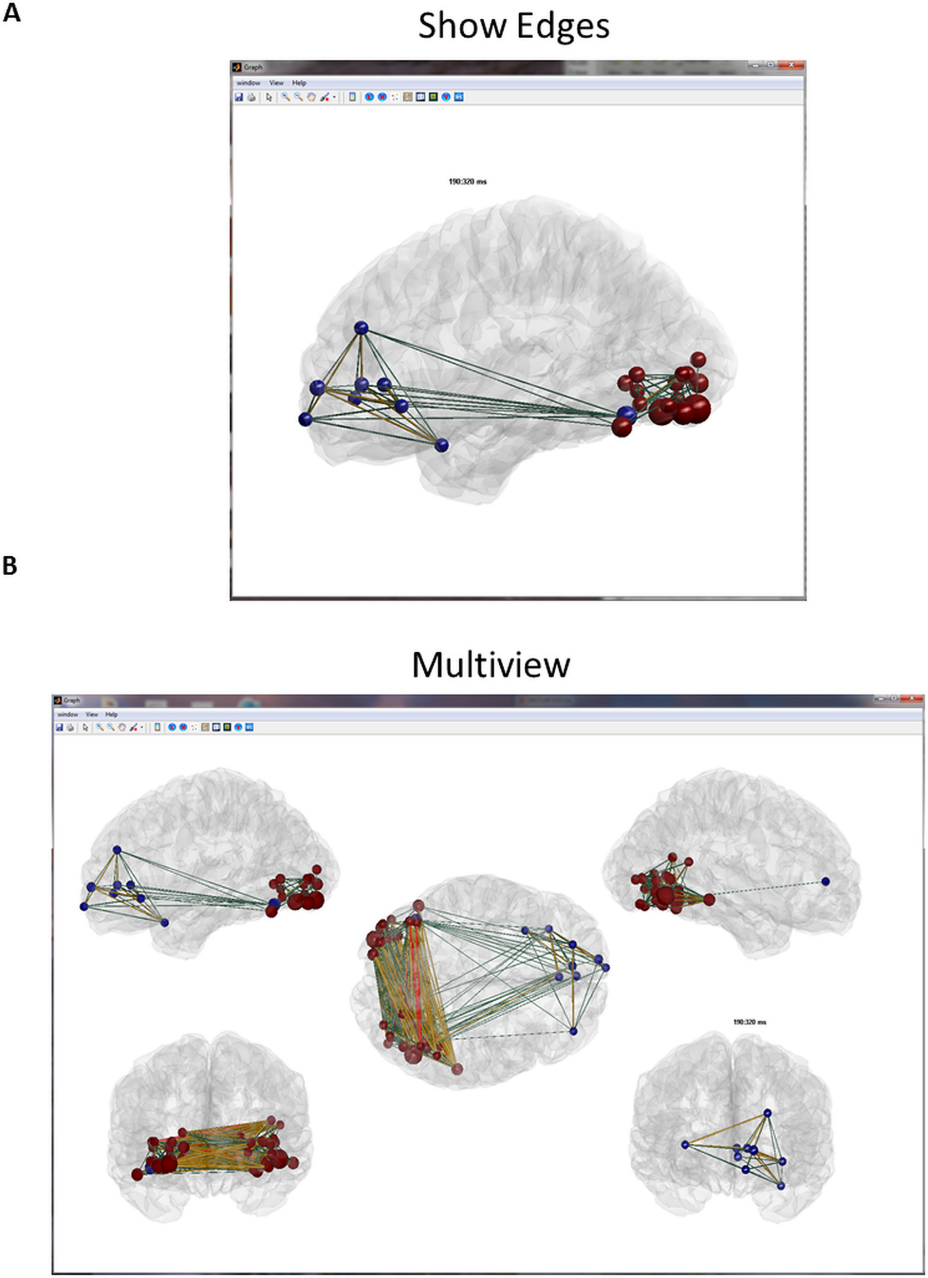
Example of the edges visualization control. A. Edge’s color represents strength values B. Multiview option (5 views) selected from the control panel (bottom).

This step can be performed within EEGNET or elsewhere (in Brainstorm for instance). The file containing the time series of the sources can be directly loaded as input for the next step, which consists in computing the functional connectivity, see [3] for comparison of several inverse algorithms and connectivity measures. In addition, the user can directly import the connectivity matrix computed elsewhere (see *Examples* folder for an example of source level matrix). Once the connectivity matrices are obtained and loaded, a set of measures can be extracted from these matrices. We integrated a number of network measures developed in the BCT toolbox [31]. EEGNET also provides the possibility of interacting with the different calculated network measures such as controlling the size and color of node (Fig 3) and edges (Fig 4). Figs 3 and 4 are typical examples representing the network obtained during picture naming task at 190ms-320ms segmented using *k-means* clustering tool of the functional connectivity [47].

First, Fig 3A shows the visualization of nodes on the cortex—without showing the edges—(modules are color-coded). As depicted, the obtained network contains two main modules. The thresholded network indicates the presence of two main modules. The first ones contain a nodes located in the bilateral occipital region and the second module is mainly located in the left frontal lobe. Second, Fig 3B shows the left view of the same network. It shows also the possibility for the user to show the corresponding atlas.

Fig 4 displays the same network as the one showed in Fig 3 but with the addition of the edges. The edges can be also coded in color and size. In Fig 4A, the color represents the weight. Three options are available for coding the edges: i) show all edges with the same color, ii) using a specific color-map or iii) coding the edges in three different colors normalized to the highest weight values.

Fig 4B shows the ‘multiview’ option that consists in showing the network from different views in the same figure. Different ‘multiview’ options are available. The user has also the option to customize his ‘multiview’ by selecting the desired views from the control panel.

### Quantification

**Fig 5:**
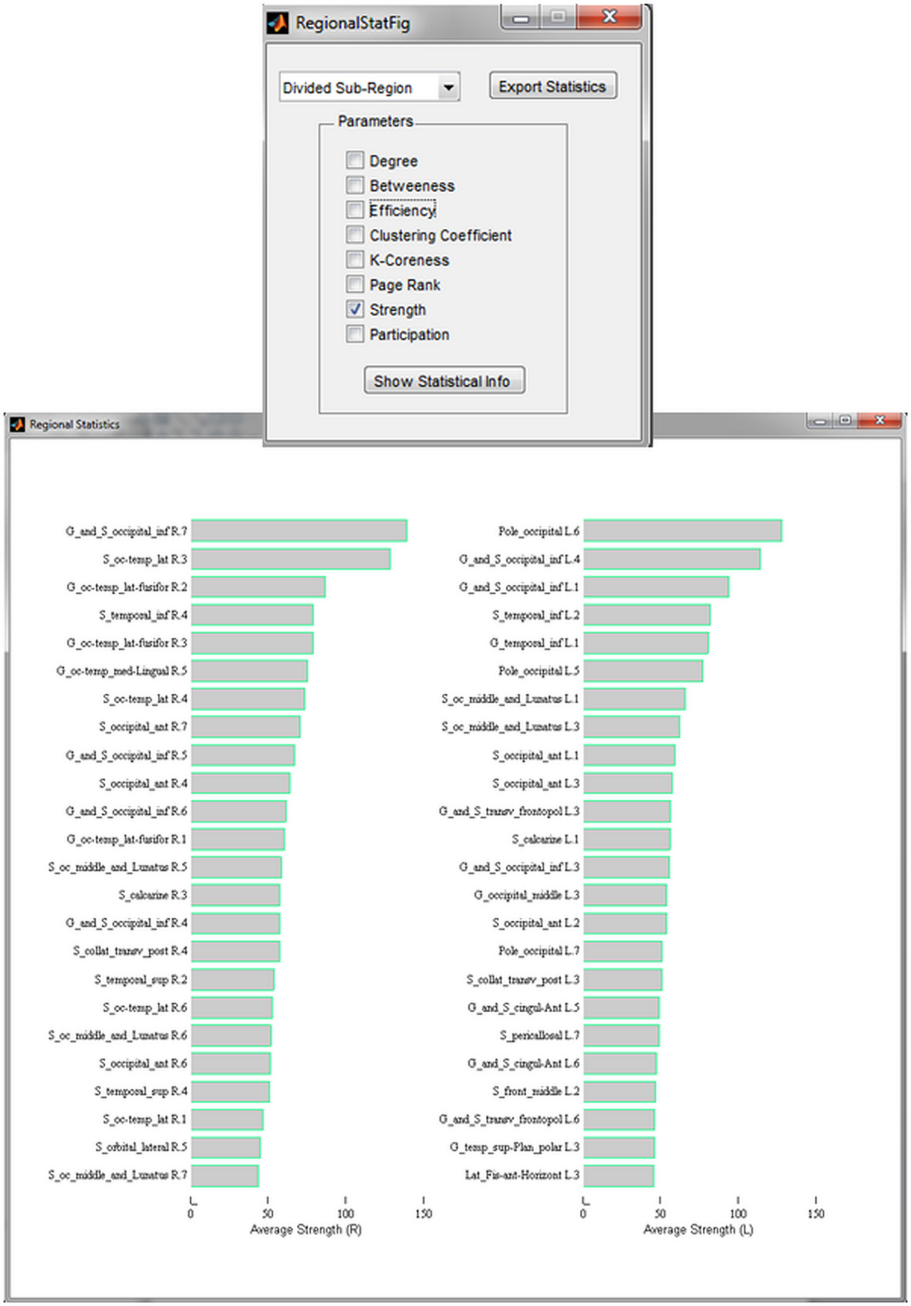
An example of the quantification analysis. The strength measure is selected. The strength values for each ROIs in the left (L) and right (R) hemispheres are showed. The labels of the ROIs are used from the already loaded scout file.

An essential feature of EEGNET is the possibility of quantifying the obtained networks. The network measures computed from the BCT toolbox can be visualized in a quantitative way as shown in Fig 5. The figure shows the results of the strength values of the different ROIs in the left and right hemispheres. It shows that the main ROIs involved in the network are the occipital regions such as occipital pole in the left hemisphere and the inferior occipital in the right hemisphere. Other features can be also chosen such as the efficiency, the degree, the clustering coefficient or any other desired measure. It requires only the selection of the measure in the control panel showed in Fig 5.

The quantification can be realized in different level. It can show the averaged values over the brain lobe (occipital, parietal, temporal, central and frontal), the averaged values over the ROIs segmented from a given Atlas such as the 148 of Destrieux Atlas or/and the values of each subdivided ROI as shown in Fig 5 where the Destrieux Atlas was segmented into ~1000 ROIs. These values can be also exported to excel file containing the label of the ROIs and the values of all the calculated network measures. This gives the user the choice of representing the data on his way.

### Experimental results

**Fig 6:**
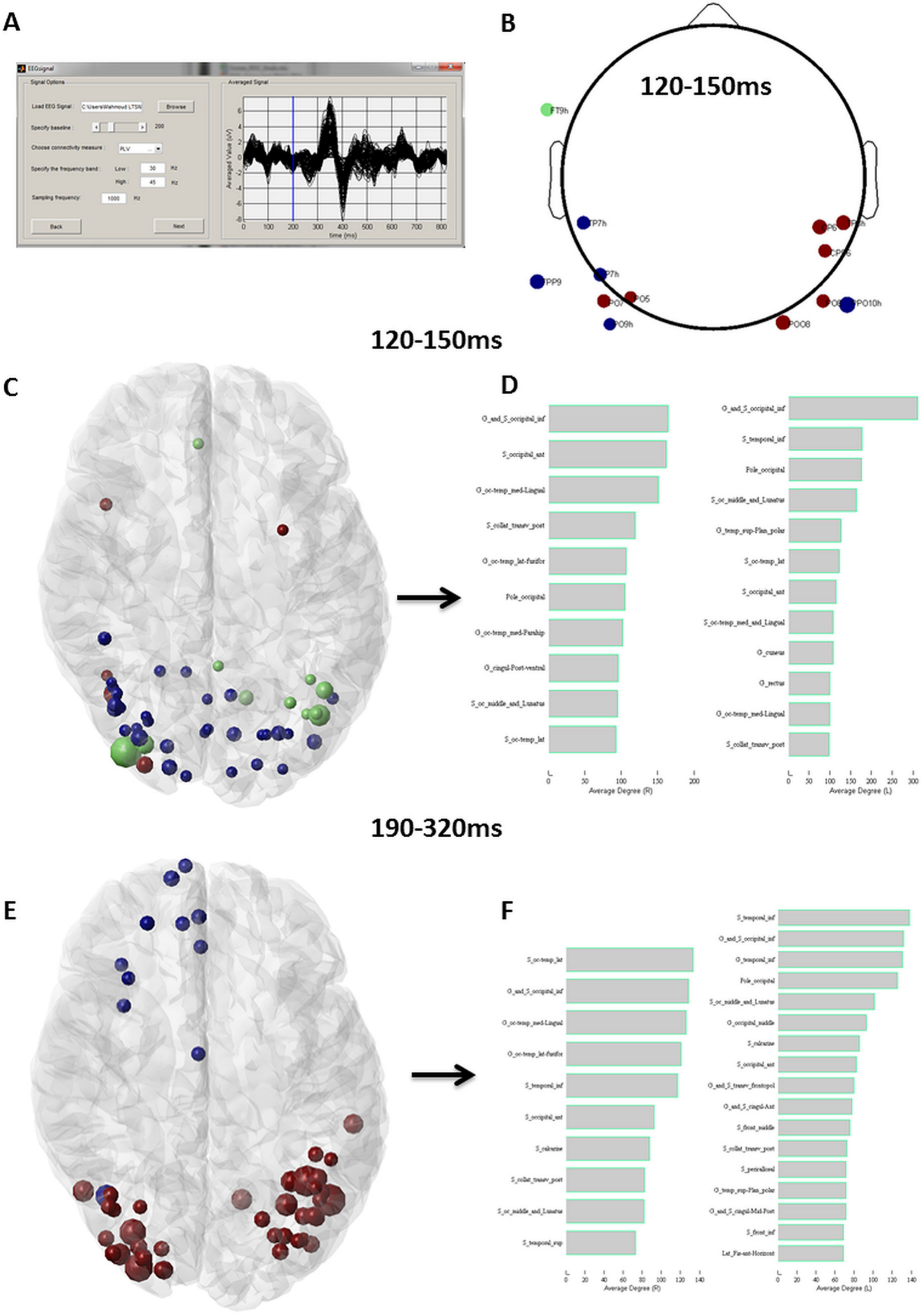
Identification of brain networks involved in picture naming task. A. The signals were loaded to EEGNET and the averaged signal over trials was visualized. The connectivity measure was chosen (PLV in this case) and the frequency bands were set to 30–45Hz (Low Gamma band). B. The network obtained at scalp level in the period 120–150ms. C. The network obtained at the same period after source reconstruction using wMNE and connectivity measurement (using PLV). D. The degree value of each nodes (based on Destrieux Atlas) was computed and visualized. E. The network obtained at 190–320ms after source reconstruction using wMNE and connectivity measurement (using PLV) and F. the corresponding degree values. Node’s color and size represent the modularity and the degree respectively. The time periods were chosen based on automatic segmentation of such cognitive task [47, 74].

In this section, we show the difference steps realized using EEGNET to identify networks involved during picture naming task for a given subject. Participant was asked to name 148 displayed pictures on a screen. The brain activity was recorded using dense-EEG, 256 electrodes, system (EGI, Electrical Geodesic Inc.). EEG signals were collected with a 1 kHz sampling frequency. After loading the signal, to obtain the scalp level network, the functional connectivity was computed using PLV method at gamma band (30–45 Hz), Fig 6A. The signal shown in Fig 6A corresponds to the average signal over trials. The vertical blue line represents the onset time instant (presentation of the visual stimulus). In our case, 200ms were taken as pre-stimulus period. After computing the network measure, the node’s color and size represent the modularity and the degree respectively. The Fig 6B shows a mainly the occipital electrodes are involved in the period between 120–200ms.

The source level network at the same period is shown in Fig 6C. The quantification of this network by computing the degree for each node shows that highest values correspond to the left/right inferior occipital, right occipital anterior and occipital pole (Fig 6D). These regions are well known to play a capital role in the processing of visual information and object recognition [51, 52]. Moreover, the gamma activity in this time period was shown to marker of object recognition and binding [52, 53]. The network in another period (190–320ms) was also illustrated and the corresponding degree values (Fig 6E and 6F). The network involves the left inferior temporal gyrus in addition to the inferior temporal sulcus. These regions were stated to be in direct relation to semantic processing (Martin & Chao, 2001). It is also the time window in which the N200 classically appear. The N200 is a marker of semantic processing in go/no-go tasks (Thorpe et al., 1996). For more details about the picture naming task and the networks corresponds to different periods, see [3].

### Technical details

EEGNET was developed using MATLAB as programming language with a user-friendly GUI under 64 bit Windows 7 environment, this toolbox has been successfully tested on different operating systems with MATLAB installed, including Windows 7, Linux and Mac OS under 64-bit versions. To facilitate the first use of EEGNET, tutorial and user manual documents are available in the download webpage that also provides the user with some examples for scalp and source networks. It is worth mentioning that EEGNET depends on other software tools. Some of these tools are written in MATLAB such as the BCT toolbox [31]. Preferably, the cortical surfaces and the scout files may be generated using Freesurfer [54] and checked/visualized using Brainstorm [30].

### Software License

EEGNET is licensed under the GNU General Public License version 1. This is a free software license, such that EEGNET may be freely redistributed and modified by any party. However, when distributing the software, the imposition of any restrictions on any further redistribution is forbidden.

### Data

The sample data used in the paper was approved by the National Ethics Committee for the Protection of Persons (CPP), *conneXion* study, agreement number (2012-A01227-36), promoter: Rennes University Hospital, Rennes, France. Participants provided their written informed consent to participate in the study.

## Discussion

To identify networks from M/EEG data, at least four tools are required from loading/preprocessing the EEG data, solving the inverse problem, computing the functional connectivity, computing the network measures to then visualizing the identified networks in interactive way. However, researchers always look for reducing the number of tools they use to accomplish a complete data processing. As a network identification/visualization tool, EEGNET achieves most of these functions.

In addition, the interactive analysis/visualization is a crucial part of scientific research. The easy visualization of data can inspire novel hypotheses, help researchers to quickly evaluate their results, and allow for significant quality control. EEGNET has novel interactive visualization features not available in existing software packages for visualization of the connectome such as the ability to interactively threshold networks based on the network measures for instance.

### Approaches for data collecting

EEGNET provides all the steps from loading the EEG signals to the identification of the brain networks. However different files are needed to accomplish these steps. Here we show some suggestions to how obtain these files.

#### A. The cortical surface

This file is very crucial to the analysis/visualization of the cortex level networks. Using the structural MRI of the participant (or template), FreeSurfer [54] can be used to compute all the different cortical parcellation with the correspondent different atlases. Performing all the cortical reconstruction steps, including subcortical segmentation for both hemispheres may take about ~16h for each MRI. The Destrieux and Desikan reconstructions atlases [45, 46] can be generated automatically using FreeSurfer and divide the cortical surface into parcels based on macroscopic sulcal and gyral profiles. The parcellation can be further subdivided into finer regions in order to generate for instance ~1000 regions (see [3, 10]). This parcellation can be realized/visualized in Brainstorm [30] and related.mat file could be exported. After choosing the desired spatial resolution (number of ROIs), the scout file contains the position and the label of each of the ROIs can be also exported. This exported file can be then used in EEGNET.

#### B. The functional connectivity matrices

The functional connectome is characterized by statistical independences between neural activities in different regions. In the M/EEG context, the FC is usually computed between signals recorded at the scalp signals using different methods such as cross-correlation [55, 56], phase locking value [44], nonlinear correlation coefficient [57, 58], phase lag index [59], imaginary coherence [60], mutual information [61] and others (see [43] for review). This can be realized in resting states or evoked activities. MNE python [62], Brainstorm [30], Brainwave (http://home.kpn.nl/stam7883/brainwave.html) and the MATLAB toolbox for FC [63] are open-source software packages with the ability to calculate many FC metrics from M/EEG data.

### Comparison between EEGNET and existing tools

In fact, EEGNET makes use of one third-party toolbox, namely the Brain Connectivity Toolbox (BCT). BCT provides tools for network analysis based on graph theory; It was interfaced with EEGNET such that measures on networks can be immediately computed without any interaction with the BCT codes. However EEGNET goes beyond the sole computation, as we added a unique feature allowing the user to visualize the BCT measures as “intuitive” graphical features related to network nodes (for instance the sphere radius/color) and edges (for instance the thickness/color).

EEGNET and the exiting M/EEG toolboxes (such as EEGLAB or Brainstorm) respond to distinct (and somewhat complementary) objectives. The main originality of EEGNET is to provide functional connectivity measures (phase synchronization, mutual information,…), 2D/3D connectivity visualization nor network measures (via BCT, based on graph theory). In the latest version of brainstorm, some connectivity measures (with circular visualization) were included. However, it does not implement EEG scalp connectivity visualization or EEG source connectivity 3D visualization. Moreover, network measures were not included in brainstorm.

Freesurfer is open-source software widely used by the Neuroimaging community. It was not integrated with EEGNET but can be optionally used to get the cortical surface from MRI in the case where subject-specific data is necessary. The corollary is that users interested in scalp EEG connectivity do not need to use Freesurfer. Users interested in the EEG connectivity at the source level will need to load their own cortical surface or use the template provided in EEGNET.

eConnectome [37] is the closest tool to EEGNET. Although both tools have the same objective of identifying and analyzing networks from M/EEG data, including preprocessing, solving of inverse problem, connectivity analysis and network visualizing, several considerable differences between EEGNET and eConnectome can be addressed:

#### i. Network measures

A crucial difference between eConnectome and EEGNET is that EEGNET offers the possibility of computing the network measures (graph theory based analysis) directly on the graph. This step became crucial when analyzing functional brain networks [64]. With EEGNET, the topological property of the networks can be investigated from global features such as density, modularity and small-worldness to more node specific properties such as degree, strength, clustering coefficient, shortest path length and edge property such as edge betweenness, shortcut edges and edges neighborhood overlap.

#### ii. Connectivity measures

The two tools include totally different family of connectivity measures. The current version of EEGNET provides four different functional connectivity measures, the cross-correlation, the mean phase coherence (MPC), the mutual information (MI) and the Phase Locking Value (PLV) while eConnectome offers methods of functional connectivity based on the multivariate autoregressive mode (mainly Directed transfer function DTF and adaptive DTF).

#### iii. Visualization

EEGNET also provides the possibility of visualizing the network based on the computed metrics. Therefore, the visualization is very different between eConnectome and EEGNET. In EEGNET, the nodes and edges can encode the network measures providing the user with direct and intuitive representation of graph features. For instance, the node size and color can be changed to render the degree, strength, modules or any other nod’s features. Similarly, the edge size and color can represent the weights for instance.

On the other side, EEGNET is restricted to M/EEG signals while eConnectome offers the possibility of analyzing functional networks from ElectroCorticographic (ECoG) data.

### Applications

Many studies reported that scalp magneto/electro-encephalography (M/EEG) connectivity may bring relevant information for example about disrupted functional networks associated epilepsy [65] or with tumors [1]. Yet, the interpretation of connectivity measures from sensor level recordings is not straightforward, as these recordings suffer from a low spatial resolution and are severely corrupted by effects of field spread [4]. For this reason, the past years have witnessed a noticeable increase of interest for functional connectivity at the level of brain sources reconstructed from M/EEG scalp signals. This approach is conceptually very appealing as networks are directly identified in the source space, typically in the neocortex. The advantage is that this approach provides an excellent temporal and very good spatial resolution [3, 4]. This method involves two main steps: i) solving the M/EEG inverse problem to estimate the cortical sources and reconstruct their temporal dynamics and ii) measuring the functional connectivity to assess statistically significant relationships among the temporal dynamics of sources. Several studies showed the usefulness of this technique mainly in brain disorder context such as the epilepsy [66–68]. However, it became trivial to characterize brain networks using approaches based on the graph theory [64].

In this context, EEGNET provides the unique tool that combines the functional connectivity analysis from EEG data with the possibility of characterizing the networks using graph theory based analysis. This possibility of computing the network measures in EEGNET is in great interest for different application such as detecting disrupted nodes/edges properties during brain disorders.

### Limitations and future directions

Further ways for software improvements may include the use of new visualization approaches or improve the existing ones. For instance, EEGNET will be updated to visualize modular partitions of brain networks, allowing for comparisons to well-studied brain networks (e.g., default mode network). The circular view of the brain network used in different tools such as CVU will be also included in EEGNET. Analyzing the dynamics of the identified networks is an important direction of future work, by including algorithms for functional connectivity states for instance [47].

The current version of EEGNET does not provide all preprocessing features. Different preprocessing modules will be included in the next version of EEGNET such as bad channel/trials and artifact removal [69]. EEGENT will be improved to support also the different M/EEG format/devices.

In the current version of EEGNET, the analysis can be realized on a single subject or on averaged data. In the next version, group analysis will be included in order to study the inter-subject variability and the possible difference between subjects or/and conditions. Concerning the inverse problem algorithms, three different algorithms are integrated in the current version: the Minimum Norm Estimate (MNE), the weighted MNE and the Low resolution Brain Electromagnetic Tomography (LORETA). Descriptions about these methods can be found in [3]. Other algorithms are also expected to be included such as MUSIC-based algorithms [70], the beam-forming algorithm [71] or algorithms based on the maximum entropy [72, 73]. Concerning the connectivity measures, we also expect to add other methods in the next version mainly the effective connectivity methods. Note the EEGNET in its current version is supporting the effective representation (using arrows indicating the direction of the connectivity). In the context of effective connectivity, eConnectome can be the best alternative to use [37].

## Conclusion

We have developed a new software tool called EEGNET. The main objective of this tool is to cover the complete processing framework from the M/EEG pre-processing to the identification of the functional brain networks. EEGNET includes mainly the calculation of the functional connectivity between scalp M/EEG signals as well between reconstructed brain sources obtained from the solution of the inverse problem. It also includes the characterization of the brain networks by computing the network measures proposed in the field of graph theory. EEGNET provides user-friendly interactive 2D /3D brain networks visualization.
